# An Unusual Presentation of Multiple Sclerosis in a Middle-Aged Woman: A Case Report and Literature Review

**DOI:** 10.7759/cureus.11017

**Published:** 2020-10-18

**Authors:** Saurabh Kataria, Karun Neupane, Zahoor Ahmed, Usama Rehman, Saba Asif

**Affiliations:** 1 Neurology and Neurocritical Care, University of Missouri Columbia, Columbia, USA; 2 Internal Medicine, Manipal College of Medical Sciences, Pokhara, NPL; 3 Internal Medicine, King Edward Medical University, Lahore, PAK; 4 Anaesthesia, Mayo Hospital, Lahore, PAK; 5 Internal Medicine, University at Buffalo/Sisters of Charity Hospital, Buffalo, USA

**Keywords:** multiple sclerosis, itching, pruritus, sensory

## Abstract

An intense itching localized to dermatomes is a rare symptom of multiple sclerosis (MS). Herein, we report a case of a 45-year-old female who presented with severe itching and tingling sensation, gait disturbance, and bilateral paresthesia for one week. She also had a history of multiple admission in the hospital due to recurrent walking abnormalities and numbness and tingling of both hands associated with intermittent psychiatric symptoms. The neurological examination revealed spastic quadriparesis with lower limb muscles affected more than the upper limbs, numbness, and sensory loss in the upper extremities in the glove and stocking pattern. Magnetic resonance imaging (MRI) revealed multiple small rounded periventricular plaques in both hemispheres and along the long axis of the corpus callosum (fluid-attenuated inversion recovery/FLAIR sequence), and cerebrospinal fluid analysis revealed the presence of oligoclonal bands, suggestive of MS. She was commenced on methylprednisolone and carbamazepine, leading to progressive resolution of her signs and symptoms. She was discharged with monthly natalizumab, and she was doing well on her follow-up.

## Introduction

Sensory symptoms are one of the most common presenting symptoms of multiple sclerosis (MS). The common presenting symptoms of MS include sensory symptoms in limbs (31%), visual loss (16%), motor symptoms (13%), diplopia (7%), gait disturbances (5%), balance problems (3%), sensory symptoms in the face (3%), Lhermitte sign (2%), vertigo (2%), etc. [1].

Sensory symptoms are classified under the positive symptoms and these can be persistent or paroxysmal. These comprise radicular pain, numbness, coldness, tightness, tingling, and pins and needle sensations [1]. An intense itching sensation localized to dermatomes and upper part of the body is a rare symptom seen in MS. Itching in lower extremities is even less reported than in the upper part of the body. Here, we report a case of a female patient with MS who presented with intense itching in the lower extremities.

## Case presentation

A 45-year-old female with a history of diabetes mellitus presented with complaints of episodic intense itching and tingling sensation on the lower half of the body for one week. These episodes began and ended suddenly within a few seconds. She also complained of difficulty in walking and bilateral prickling sensation in the upper limbs for the last week, following the recent flu. She also has a history of multiple admissions in the hospital due to recurrent walking abnormalities and numbness and tingling of both hands. These symptoms were resolved after a few days and she experienced similar symptoms in lower parts of the body. She also reported intermittent psychiatric symptoms including severe fatigue, aggression, and loss of energy for the last two years. She also had a history of falls following the signs and symptoms. There was no history of trauma, cough, or contact with a person with a chronic cough, and no band like sensation. She is not an alcoholic and does not smoke.

Initial evaluation revealed a temperature of 98 °F, blood pressure of 110/80 mmHg, heart rate of 95 beats per minute, respiratory rate of 19 per minute, and oxygen saturation of 98% on room air. On an extensive neurological examination, she had spastic quadriparesis with lower limb muscles affected more than the upper limbs. The general sensations were reduced in the lower limbs. However, numbness and sensory loss were noted in the glove and stocking pattern in the upper extremities. There was no sign of meningeal irritation or cranial nerve deficit. On the psychopathological examination, the patient exhibited reduced incitement and a slow thought process.

**Figure 1:**
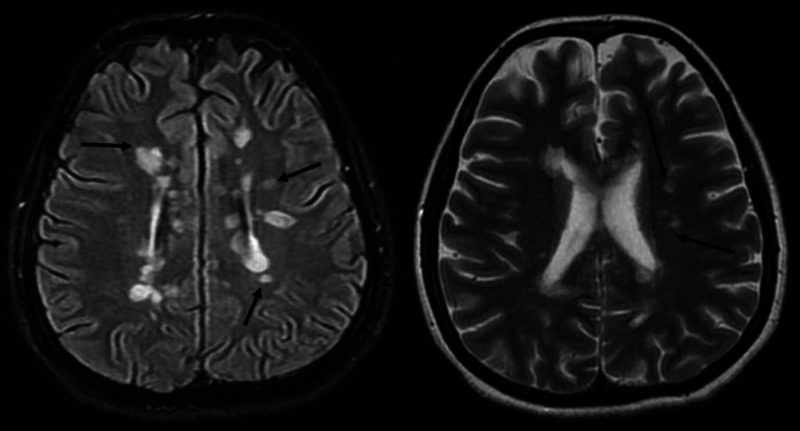
Axial FLAIR T-2 weighted images of the patient’s hemisphere, showing multiple hypointense and hyperintense periventricular lesions, typical of demyelination (black arrows). FLAIR: Fluid attenuated inversion recovery.

**Figure 2:**
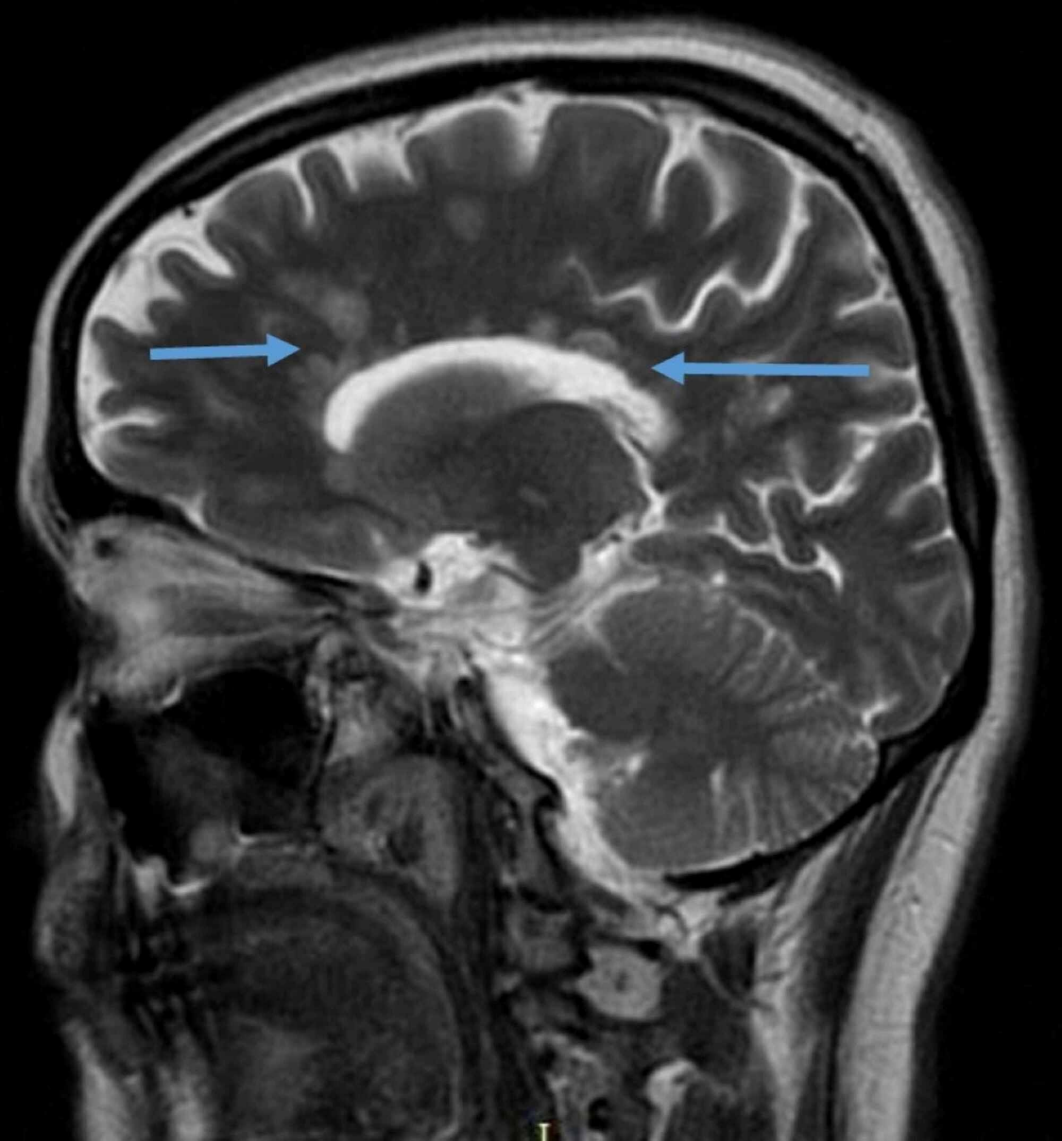
Coronal FLAIR T-2 weighted image of the patient’s brain demonstrating multiple periventricular plaques with long axis perpendicular to the corpus callosum (blue arrows). FLAIR: Fluid attenuated inversion recovery.

The symptoms were suggestive of central brain lesions, and magnetic resonance imaging (MRI) was performed, which revealed multiple small rounded periventricular plaques in both hemisphere and along with the long axis of the corpus callosum (fluid-attenuated inversion recovery/FLAIR sequence) and multiple fresh inflammatory areas after gadolinium enhancement (Figures 1 and 2). MRI of the spine was normal. On further workup, the patient was negative for hepatitis B virus, human immunodeficiency virus, syphilis, hepatitis C virus, autoimmune antibodies including antinuclear antibody, double-strand DNA antibody, phospholipid antibody, antineutrophil cytoplasmic antibody, perinuclear antineutrophil cytoplasmic antibody, and paraneoplastic syndrome with normal erythrocyte sedimentation rate and C-reactive protein. The cerebrospinal fluid analysis revealed positive oligoclonal bands, suggestive of MS diagnosis.

She was started on methylprednisolone 2 g and carbamazepine which led to gradual resolution of her signs and symptoms. She was discharged with monthly natalizumab and she was doing well on her follow-up.

## Discussion

Paroxysmal itching is one of the positive symptoms in patients with MS [2]. Paroxysmal itching as an early symptom of MS has been previously reported by Sakurai and Kanazawa [2], Yamamoto et al. [3], Koeppel et al. [4], Osterman [5,6], Yabuki and Hayabara [7], and Sandyuk [8]. The patients in these reports complained of pruritus on dermatomal patterns on the face and trunk.

Yamamoto et al. reported a patient with MS who developed itching on lower extremities and two other patients with itching on the neck, face, and upper extremities [3]. Koeppel et al. reported a patient with itching localized to shoulders [4]. Osterman and Westerberg reported a woman with pruritus on thighs and hands [5]. Osterman reported two additional cases with pruritus on the upper extremities and back [6]. Yabuki and Hayabara reported a case with pruritus on the face [7]. Sandyuk reported two cases of MS who developed itching after an experimental treatment with an external magnetic field [8]. Sakurai and Kanazawa reported seven cases of MS with pain or itching. Face and chest were involved in six of them while lower extremities were involved in only one patient [2].

The characteristics of itching common to these reported cases include abrupt onset and end of the episodes ranging from several seconds to several minutes with a frequency of 5-6 times a day or more mostly including the face, trunk, and extremities and rarely lower extremities [9]. The case described here presented with symptoms similar to those described by the aforementioned reports. However, this case presented with the additional rare symptom of intense itching on the lower extremities.

Itching is considered as a form of subthreshold pain. It has been hypothesized that one of the reasons for the attacks of paroxysmal itching is increased temperature, and hence, these attacks have been seen while taking a hot bath or during sleep when the body temperature increases by 0.5-1.0 °C [3]. The stimuli responsible for itching create impulses that are carried by slow conducting unmyelinated C fibers to the spinal cord via posterior roots which are in turn are carried by the pain fibers to the thalamus [3].

Similar to the previously reported cases, pruritus in this patient responded to carbamazepine. The patient responded well to the treatment with carbamazepine, methylprednisolone, and natalizumab.

## Conclusions

Severe itching can be a part of the constellation of presenting symptoms of MS, though it is highly uncommon. MS should be considered as a differential diagnosis for any patient who presents with intermittent lower and upper motor signs and symptoms separated in time and place. Due to its variable clinical presentations, a high index of clinical suspicion is required for the diagnosis of MS. Therefore, clinicians should have sound knowledge and pay close attention to MS and its related complications.
